# A mixed-methods analysis of risk-reduction strategies adopted by syringe services program participants and non-syringe services program participants in New York City

**DOI:** 10.1186/s12954-023-00772-3

**Published:** 2023-03-25

**Authors:** Nisha Beharie, Adelya Urmanche, Alex Harocopos

**Affiliations:** New York City Department of Health and Mental Hygiene, 42-09 28th Street, Long Island City, NY 11101 USA

**Keywords:** People who use drugs, Syringe services programs, Fentanyl

## Abstract

**Background:**

Since the emergence of fentanyl in the drug market, syringe services programs (SSPs) have been at the forefront of providing life-saving tools such as naloxone and fentanyl test strips to people who use drugs (PWUD). It is still unclear, however, how the adoption of risk-reduction practices has differed among PWUD in the context of increasing presence of non-pharmaceutical fentanyl in the drug supply. This study aims to assess the adoption of risk-reduction tools (e.g., naloxone) among those engaged with SSP services and those not engaged with SSP services.

**Methods:**

We conducted a mixed-methods study following a convergent parallel design integrating both quantitative and qualitative data. Interviews were conducted with 80 people who used street opioids (i.e., heroin or opioid pills not prescribed), 32 of whom were not engaged in SSP services. Quantitative differences between those engaged and those not engaged in SSPs were assessed using independent samples *t* tests and Fisher’s exact tests. A thematic analytic approach was employed to compare qualitative responses between the two groups.

**Results:**

Three main themes emerged in our analysis: (1) Both groups expressed an interest in fentanyl test strips (FTS), but those engaged in SSP services found them to be more accessible; (2) there was greater adoption of and enthusiasm for naloxone among SSP participants; and (3) SSP participants were more likely to have or be interested in having someone check in on them when using alone, but stigma and perceived personal risk of overdose prevented widespread adoption of this practice among all participants.

**Conclusion:**

SSPs provide a vital function by facilitating naloxone and FTS distribution to participants who often have little control over their exposure to fentanyl. However, stigma and misconceptions regarding drug use are barriers to people adopting risk-reduction practices, particularly among those not engaged with SSPs.

## Introduction

Overdose mortality continues to be a critical public health concern, with recent provisional data showing that 81,230 drug overdose deaths occurred in the USA in the 12 months ending in May 2020 [1]. These data represent a continuing upward trend over the previous decade and the largest number of drug overdoses for a 12-month period ever recorded. The rise in drug-related mortality is driven largely by the proliferation of non-pharmaceutical fentanyl (NPF) and NPF analogs in the illicit market, reports of which have increased significantly across the country from 2009 to 2019 [2]. Estimated to be 50–100 times stronger than morphine and cheaper than heroin, NPF enters the illicit drug supply primarily as a heroin adulterant [3], though it has also been found in other substances. In New York City (NYC) there were 2668 overdose deaths in 2021, 80% of which involved fentanyl [4]. For the fifth year in a row, fentanyl was the most common substance involved in overdose deaths.

In response to the increase in fentanyl-involved overdoses, syringe services programs (SSPs) across NYC have taken steps to provide risk-reduction strategies to people who use drugs (PWUD). SSPs are community-based harm reduction programs for PWUD, which have shown to be effective at reducing the transmission of HIV and hepatitis as well as preventing overdose deaths [5]. In NYC, there are 15 SSPs, providing services via 14 static locations, 33 mobile sites, and peer-delivered syringe exchange (PDSE). In addition to offering sterile drug use and safe sex supplies, hepatitis screening, and healthcare coordination services, SSPs in NYC offer services such as overdose prevention training with take-home naloxone, and more recently, in response to the emergence of fentanyl in the drug market, fentanyl test strips (FTS) [6, 7]. FTS were originally developed to test for the presence of fentanyl in urine. However, more recently, they have been used off-label to test for the presence of fentanyl in drugs and drug residue [6].

According to McAteer et al. [8], however, the most common service received by SSP participants in NYC in 2018 was health education and promotion. In addition to overdose prevention training mentioned above, SSPs encourage individuals to use with other people, or to call someone prior to using alone who could then alert first responders or respond themselves if they believe the individual may be overdosing. Commonly referred to as a check-in plan, this strategy is being promoted largely in response to the potency of fentanyl and the speed with which it takes effect. Other risk-reduction strategies include testing the potency of a drug by using a small amount and using the drug slowly [9].

The current literature has outlined the response among PWUD to some of the risk-reduction strategies that have been promoted due to the increased prevalence of NPF in the illicit drug market. Mars and colleagues [10, 11], for example, found that some PWUD were using smaller drug doses to gauge potency, while others were more likely to carry naloxone and/or use it with other people to mitigate the risk of fatal overdose. Similarly, Carroll et al. [12] found that, in addition to using smaller quantities, PWUD were employing other methods to reduce their risk of overdose, such as relying on a consistent source for drugs or using prescription opioids instead of heroin. Most recently, and specific to NYC, McKnight and Des Jarlais [13] reported similar trends in fentanyl adaptation among PWUD including increased utilization of “test shots” (i.e., shots with a small amount of drugs), naloxone, and FTS, as well as using with others and maintaining a consistent source.

Methodologically qualitative, these studies were almost exclusively comprised of participants engaged in SSP services, and involving PWUD in research who are not connected to services continues to be a challenge [14]. The previous literature suggests that SSP engagement is associated with reductions in high-risk drug use behaviors, particularly syringe-related behaviors (e.g., sharing syringes, reusing syringes) [15, 16]. At the same time, it is still unclear how the adoption of risk-reduction practices has differed among SSP participants and those not engaged in SSP services, during this time of increasing presence of NPF in the drug supply. This study adds to the extant literature by including PWUD who are not accessing SSP services and taking a mixed-methods approach. The aim of the analysis was to assess the extent to which there were differences in the adoption of strategies to prevent fatal overdoses among those who accessed SSP services and those who did not.

## Methods

### Overview and design

FASt (Fentanyl Adaptation Study) consisted of a mixed-methods, cross-sectional study of people who use street opioids (i.e., heroin, or opioid pain pills not prescribed) in NYC. All study procedures were approved by the Institutional Review Board at the NYC Department of Health and Mental Hygiene and verbal informed consent was obtained for all participants. The interview guide was informed by ten key informant interviews with peer advocates at five syringe services programs (SSPs) across the city. Questions from the key informant guide focused on how PWUD had responded to the presence of fentanyl in the drug supply. The key informants also served as an initial referral source for participants included in the study.

### Procedures

#### Recruitment

Participants were recruited through a combination of convenience sampling from five SSPs, street-based recruitment (e.g., approaching individuals engaging in public drug use, flyers on light posts), as well as snowball sampling. Potential participants were provided with a brief study description and interested parties were directed to contact a FASt staff member to complete the screening and determine eligibility for the study. Inclusion criteria were: (1) age 18 years or older; (2) used street opioids (i.e., heroin or opioid pain pills not prescribed) at least twice during the past month; (3) were aware of NPF in the illicit drug supply; and (4) resided in NYC or bought and/or used drugs primarily in NYC. Participants who completed the interview were asked to refer up to three individuals to facilitate snowball sampling. Participants received a $50 honorarium for their time. Participants were also eligible to receive a further honorarium of $20 each for each referred participant who completed an interview. All participants were offered a naloxone kit at the conclusion of the interview.

#### Data collection

Data collection occurred from March 2018 to August 2019. Most interviews took place in public and semi-public venues (e.g., fast food restaurants, coffee shops, and parks). The interview guide was semi-structured and consisted of both open-ended questions as well as closed-ended items which covered the participant’s: (1) demographic characteristics; (2) knowledge of and response to fentanyl; (3) drug purchasing practices (e.g., how many people they buy from, whether they find them reliable); (4) experience with fentanyl test strips; (5) experience with overdose and naloxone; (6) current drug use; and (7) opinions of health messaging related to fentanyl. Responses to closed-ended questions were collected on a paper interview form. All interviews were recorded and professionally transcribed and lasted an average of 65 min (range 35–120 min).

The specific phrasing of closed-ended items can be found in Table 1 through Table 4. The race variable in the table was a combination of a variable measuring ethnicity (i.e., Latinx vs. non-Latinx) and race (i.e., Black, White, Asian, multiracial, other) and includes those racial categories endorsed by the respondents. The item related to injection drug use with opioids was captured by asking the participant the route of administration for each drug they had used in the past 30 days (e.g., sniffing, smoking, injecting). The responses for opioids were then collapsed into a dichotomous variable, “injection,” and “intranasal.” Only data relevant to the focus of this manuscript are reported in the results and discussion section. Participants were asked to create their own pseudonyms in order to protect their anonymity. These pseudonyms are used when reporting direct quotes.

**Table 1:** Demographics

	Non-SSP (*N* = 32)	SSP (*N* = 48)	Total (*N* = 80)	
	Mean (SD)			*t* test
Age	40.9 (11.4)	41.2 (11.1)	41.1 (11.1)	*t*(78) = − 0.10 *p* = 0.922

	% (*N*)			*p* value (*X*^2^)
*“What is your gender?”*				
Male	68.8% (22)	68.8% (33)	68.8% (55)	0.662
Female	31.3% (10)	27.1% (13)	28.7% (23)	
Transgender (MTF)	0.0% (0)	4.2% (2)	2.5% (2)	
*“Do you think of yourself as straight, bisexual, gay, or something else?”*^*a*^				
Straight/Heterosexual	90.6% (29)	91.5% (43)	91.1% (72)	0.461
Gay/Lesbian/Homosexual	0.0% (0)	4.3% (2)	2.5% (2)	
Bisexual	9.4% (3)	4.3% (2)	6.3% (5)	
*“Which one or more of the following would you say is your race?” and “Are you of Hispanic/Latinx origin?”*^*a*^				
White (non-Latinx)	21.9% (7)	31.9% (15)	27.8% (22)	0.055
Black (non-Latinx)	37.5% (12)	12.8% (6)	22.8% (18)	
Latinx	28.1% (9)	42.5% (20)	36.8% (29)	
Multiracial	6.3% (2)	12.8% (6)	10.1% (8)	
Other	6.3% (2)	0.0% (0)	2.5% (2)	
*What is the highest level of education you have reached?*				
Did not complete high school	25.0% (8)	14.6% (7)	18.8% (15)	0.043
Completed high school/GED	28.1% (9)	45.9% (22)	38.8% (31)	
Some college	18.8% (6)	33.3% (16)	27.5% (22)	
Tertiary education (i.e., AS, BS, or graduate degree)	28.2% (9)	6.3% (3)	15.1% (12)	
*In the last 30 days where have you spent the majority of your nights?*				
Place I consider my own home	53.1% (17)	33.3% (16)	41.3% (33)	0.467
Family or friend’s home	9.4% (3)	20.8% (10)	16.3% (13)	
Shelter or emergency housing	25.0% (8)	27.1% (13)	26.3% (21)	
Hotel	0.0% (0)	2.1% (1)	1.3% (1)	
Street/sidewalk	9.4% (3)	6.3% (3)	7.5% (6)	
Subway/bus vehicle or station	0.0% (0)	2.1% (1)	1.3% (1)	
Other	3.1% (1)	8.3% (4)	6.3% (5)	
*Inject Heroin and/or NPF*^*b*^				
Inject	30.0% (9)	60.4% (29)	48.7% (38)	0.011
Sniff	70.0% (21)	39.6% (19)	51.3% (40)	

^a^Contains missing data (i.e., sample does not add to 80)

^b^Column percent for this category does not include “Not Applicable” (i.e., sample does not add to 80 as 2 participants had only used pain pills not prescribed orally)

### Data analysis

For this study, we took a mixed-methods analytic approach that followed the convergent parallel design in which the qualitative and quantitative methods were used to obtain triangulated results about a single topic [17]. Qualitative and quantitative data were collected concurrently but analyzed separately. Findings were then synthesized by identifying content areas represented in both the qualitative and the quantitative data and ways in which the results from both types of data converged, diverged, or produced a more complete understanding of the research topic. Responses from those who reported SSP service engagement in the previous 12 months were compared to those who had not engaged in SSP services during the same period. Study participants were considered engaged in SSP services if they described accessing SSP services during the past year from either a static (brick and mortar) site, or a mobile (van) program.

#### Quantitative

Quantitative analyses were conducted using SPSS software [18]. Descriptive statistics were calculated for those engaged in SSP services and those who were not, as well as for the total sample (see Table 1). Means and standard deviations were calculated for age and number of naloxone kits (i.e., continuous variables), and frequencies and percentages were calculated for all remaining categorical variables. Statistically significant differences between the two groups were assessed using an independent samples *t* test for continuous variables and the Fisher’s exact test for categorical variables, with the exception of two variables that were multiselect variables and for which such a test was not possible (i.e., where participants received kits, and where they kept their naloxone kits). The Fisher’s exact test was used due to small sample sizes with small cell counts [19].

#### Qualitative

Dedoose software [20] was used to code and analyze the qualitative data employing a thematic analytic approach [21, 22]. To create the codebook, four research team members independently coded four transcripts and then came to a consensus on a final list of codes and code definitions. Each interview was then coded by two research team members independently using the final code structure and discrepancies were later reconciled. The research team served as an interpretive community to develop themes from the coded excerpts and methodological rigor was maintained through an audit trail and analytic memos [23]. The qualitative analysis of subgroups was facilitated by the mixed-methods features offered in Dedoose which allow researchers to analyze qualitative data by various groups identified by quantitative variables also entered into Dedoose (i.e., those not engaged in SSP services, and those engaged in SSP services).

## Results

### Sample description

A total of 80 participants who used street opioids were recruited, 32 of whom had not accessed SSP services in the past year, and 48 who had. SSP participants in this study were largely representative of SSP participants in NYC with regard to race, gender, and age [8]. A full description of the sample can be found in Table 1.

### Both groups expressed an interest in fentanyl test strips (FTS), but those engaged in SSP services found them to be more accessible (see Table 2)

**Table 2:** Fentanyl test strips

	Non-SSP (*N* = 32)	SSP (*N* = 48)	Total (*N* = 80)	
	% (*N*)			*p* value (*X*^2^)
*Have you heard about test strips that can detect the presence of fentanyl?*				
No	75.0% (24)	22.9% (11)	43.8% (35)	0.000
Yes	25.0% (8)	77.1% (37)	56.3% (45)	
*Have you ever received/been given a test strip?* ^*a*^				
No	87.5% (7)	27.0% (10)	37.8% (17)	0.003
Yes	12.5% (1)	73.0% (27)	62.2% (28)	
*Where did you get the test strips from?* ^*a*^				
Harm reduction/Syringe services program (Static and or Van)	0.0% (0)	96.3% (26)	92.9% (26)	0.107
Friend	0.0% (0)	3.7% (1)	3.6% (1)	
Other	100% (1)	0.0% (0)	3.6% (1)	
*Have you ever used test strips to see if your drugs contain fentanyl?* ^*a*^				
No	0.0% (0)	22.2% (6)	21.4% (6)	1.000
Yes	100.0% (1)	77.8% (21)	78.6% (22)	

^a^Column percent for this category does not include “Not Applicable.” Responses to ever being given a test strip include only the 45 participants who had heard of fentanyl test strips. Similarly, responses to source of test strips and whether they had used the test strips include only the 28 participants who had received test strips

Participants who had received SSP services were more likely both to have heard of (77.1% vs. 25.0%; *p* = 0.000) and accessed FTS (73.0% vs. 12.5%; *p* = 0.003) compared to those who had not received SSP services. All but one of the SSP participants (96.3%) who had heard of FTS acquired them through their engagement with SSPs. Of the SSP participants who had received FTS, 77.8% reported having used them. Conversely, only one participant not engaged in SSP services (3.1%) reported ever using FTS, which he had received from a drug treatment program.

Qualitative data elucidated the circumstances in which participants typically used FTS. Many reported checking their drugs either when buying from a new source or receiving a new batch from a known seller. Some also reported using FTS specifically in response to changes in the appearance of the drug (e.g., color). In many instances, receiving a positive result for fentanyl led to the participant taking precautions to reduce their risk of fatal overdose. James, an SSP participant, described this phenomenon in more detail.

I test my drugs whenever I can, especially when I'm alone […]. I remember testing a heroin batch one time and it was positive for fentanyl and because of the way it looked after putting the water, it looked like it was really, really like powerful that I shouldn't do that alone. So, I remember calling a friend, and I had [naloxone] also, but I said he didn't have to bring [naloxone] because I had [naloxone]. […] I try to carry [naloxone] all the time and I have [naloxone] in my room. And I had him come over and I told him why. I told him I was scared. I don't want to use alone.

Among participants who previously had never heard of and/or accessed FTS (*n* = 52, 65%), those engaged in SSPs were more likely to state that they would use them if they were available (95.2% vs. 71.2%; *p* = 0.036), with many in both groups citing the ability to make more informed decisions as a reason for their interest. Still, over seventy percent of participants not engaged in SSP services who had not heard of and/or received FTS reported they would use test strips if they were available to them. For example, Josh, who was not engaged in SSP services, suggested that FTS would give him more agency regarding his drug use.

Because, like I said, it's good to know […]. You can't always tell because, if they do cut heroin with it, it still gives it the color and stuff but it's just a heck of a lot more potent and it would, you know…Unless you're looking to overdose, it's really not good. Because heroin, I would use, like, four to five [bags] at a time and fentanyl, I use one, maybe two if it's, you know, cut, so.

Participants who were not interested in the prospect of FTS also provided explanations for why they would not use them which included the time intensity of testing (particularly if experiencing withdrawal symptoms), having trust in people they bought drugs from to be forthright about whether their product contained fentanyl, and the inability of FTS to indicate which particular analog of fentanyl was present or to detect all analogs. In addition, some who only used intranasally expressed concern that they would be sacrificing product to conduct a test. One non-SSP participant also mentioned that they would want to use FTS prior to rather than after purchasing a product but thought it unlikely that people who sell drugs would agree to such a request. Some non-SSP participants also mentioned the cost of purchasing FTS as a barrier to their use.

### Greater adoption of and enthusiasm for naloxone among SSP participants (see Table 3)

**Table 3:** Naloxone access and overdose

	Non-SSP (*N* = 32)	SSP (*N* = 48)	Total (*N* = 80)	
	% (*N*)			*p* value (*X*^2^)
*Have you heard of a drug called naloxone/Narcan* ^*®*^ *, a medication that can be used to treat opioid overdoses?*				
No.	3.1% (1)	2.1% (1)	2.5% (2)	1.000
Yes	96.9% (31)	97.9% (47)	97.5% (78)	
*In the last 12 months have you been offered naloxone/Narcan* ^*®*^ *, often contained in a blue bag?* ^*a*^				
Yes, and I accepted a kit/s	64.5% (20)	87.2% (41)	78.2% (61)	0.019
Yes, but I did not accept a kit because I already have at least one	0.0% (0)	2.1% (1)	1.3% (1)	
Yes, but I did not accept a kit and I don’t already have one	3.2% (1)	2.1% (1)	2.6% (2)	
No	32.3% (10)	8.5% (4)	17.9% (14)	
*Where did you get your kit(s) from?* ^*a/b*^				
SSP	0.0 (0)	87.8 (36)	59.0 (36)	NA
Methadone program	10.0 (2)	9.8 (4)	9.8 (6)	
Drug treatment or detox	35.0 (7)	0.0 (0)	11.5 (7)	
Emergency department	20.0 (4)	0.0 (0)	6.6 (4)	
Friend/partner/family member	25.0 (5)	2.4 (1)	9.8 (6)	
Service provider (e.g., homeless services, reentry support, employment assistance)	15.0 (3)	2.4 (1)	6.6 (4)	
First responder (e.g., EMS, police)	10.0 (2)	2.4 (1)	4.9 (3)	
Other	20.0 (4)	22.0 (9)	21.3 (13)	
*Where do you typically keep your naloxone/Narcan* ^*®*^ * kit?* ^*a/b*^				
On person	0.0 (0)	2.4 (1)	1.6 (1)	NA
In bag	5.0 (1)	53.7 (22)	37.7 (23)	
Place of residence	90.0 (18)	68.3 (28)	75.4 (46)	
Other	10.0 (2)	17.1 (7)	14.8 (9)	
*In the last 12 months, have you experienced a drug overdose?*				
No	62.5% (20)	72.9% (35)	68.8% (55)	0.338
Yes	37.5% (12)	27.1% (13)	31.3% (25)	
*In the last 12 months, have you witnessed someone else overdose?*				
No	62.5% (20)	16.7% (8)	35.0% (28)	0.000
Yes	37.5% (12)	83.3% (40)	65.0% (52)	

	Mean (SD)			*t* test
How many kits do you currently have?	1.1 (SD = 1.2)	2.3 (SD = 2.3)	1.7 (1.9)	*t* (56) = − 2.6, *p* = 0.013

^a^Column percent for this category does not include “Not Applicable.” Responses to ever being offered a kit include only the 78 participants who had heard of fentanyl test strips. Similarly, responses to the sources of their kits and where they keep the kits include only the 61 participants who had been offered and accepted a naloxone kit

^b^Percentages do not add to 100% because participants were able to select more than one answer category

All but two participants (one SSP participant and one non-SSP participant) had heard of naloxone; however, those engaged with SSPs were more likely both to have been offered a naloxone kit in the past year (91.4% vs. 67.7%; *p* = 0.019) and to have accepted it (87.2% vs. 64.5%; *p* = 0.019). Most SSP participants reported receiving their kits from the SSP they attended (87.8%), while those not engaged in SSP services reported receiving them from a variety of sources including: substance use treatment services (other than methadone clinics) or detox (35.0%); friends, partners, or family members (25.0%); and emergency departments (20.0%). SSP participants also reported having, on average, twice as many naloxone kits as participants not engaged with SSPs (2.3 vs. 1.1 kits; *p* = 0.013).

While the likelihood of experiencing an overdose in the past year was not statistically different among the two groups, SSP participants were more likely to have witnessed an overdose in the past year (83.3% vs. 37.5%; *p* = 0.000) and to report carrying naloxone in their bag than non-SSP participants (53.7% vs. 5.1%). This greater willingness to carry naloxone and an appreciation of the benefits of doing so (e.g., could assist in an overdose occurring outside the house) was reflected in SSP participants’ narratives about overdose reversal. Joe, an SSP participant, expressed his enthusiasm regarding naloxone.

It's [in] my bag, I carry it around with me all the time. […] I've personally…I think I've saved three people and I've assisted in dozens of you know [overdoses]. I know places where there's like an overdose once or twice a week. So, I would be coming in there and restocking their supply.

Conversely, those not engaged with SSPs tended to be less enthusiastic about naloxone and felt that, while the medication was a useful tool, it was not personally relevant to them (e.g., they did not spend time with other people who used drugs, were trying to stop using, did not “abuse” drugs). For example, Nutty, who was not engaged in SSP services, did not think he needed naloxone as he was planning to reduce his drug use by starting on a methadone program:

Because, right now, I’m getting ready to get on a methadone program. So, once I get on that, I know it’s- I’m not going to- I’m going to try not to do heroin no more.

Additionally, some participants in this group not only thought naloxone was irrelevant but were actually opposed to carrying it. For example, the fear of experiencing opioid withdrawal following the administration of naloxone led some to not want to have it on their person. Tom, a non-SSP participant who was prescribed methadone described his reluctance to carry naloxone because of his concern that, should naloxone be administered to him unnecessarily, he would go into precipitous withdrawal.

I’m not going to carry naloxone in my pocket. […] It’s really hard for someone to go from having opiates in their system to withdrawal. I wouldn’t want for me to fall out from an asthma attack and you see naloxone in my pocket and give me naloxone. And I go straight into withdrawal right here? […] I wouldn’t sleep with that thing under my pillow. It’s too much of a scary device. […] Imagine me going into straight withdrawal? Oh! That is just something I don’t even want to deal with. […] If, God forbid, I’m on the street and I really needed it, but I don’t need no Lone Ranger coming around and, “Oh, he has naloxone.” and shooting it up my nose.

### SSP participants were more likely to have and be interested in having someone check in on them when using alone, but stigma and perceived personal risk of overdose prevented widespread adoption of the practice among all participants (see Table 4)

**Table 4:** Other drug use practices

*In the last 30 days did you use drugs:*				
By myself only	37.5% (12)	31.3% (15)	33.8% (27)	0.410
With others only	15.6% (5)	8.3% (4)	11.3% (9)	
By myself and with others	46.9% (15)	60.4% (29)	55.0% (44)	
*In the last 30 days, when using by yourself, did you ever have a plan for someone to check on you in case you overdosed?* ^*a*^				
No	77.8% (21)	56.8% (25)	64.8% (46)	0.075
Yes	22.2% (6)	43.2% (19)	35.2% (25)	

^a^Column percent for this category does not include “Not Applicable.” Responses to having employed a check-in plan include only the 71 participants who had used alone in the past month

Most participants in both the non-SSP and SSP groups had used alone at least once in the previous month (84.4% and 91.7%, respectively), indicating a need for participants to implement a check-in plan. However, those connected with SSPs who had used alone were more likely to have reported doing so than non-SSP participants who had used alone (43.2% vs. 22.2% *p* = 0.075), although neither group had consistently embraced this risk-reduction strategy and the difference was not statistically significant.

Participants in both groups reported similar reasons as to why they had not instituted a check-in plan in the past month, the most common being that they did not feel they were at risk of overdose. This was typically attributed to knowing their “limit” and being confident they could control the amount they used. Others felt check-in plans were unnecessary because they believed their drug use behaviors (e.g., using only a small amount at one time, using less frequently) were protective. This lack of risk perception was particularly common among those who only used intranasally. Some participants also reasoned they did not need a check-in plan because they used it in a place where they would be easily found by friends, family, or bystanders who could assist them if the need arose.

However, one of the more pervasive reasons given by participants in both groups for not instituting a check-in plan was related to the shame and stigma they experienced regarding their drug use. In addition to not believing she was at risk of overdose, Erin, an SSP participant, also did not feel comfortable sharing with people that she used drugs and, as a result, felt check-in plans were an impractical strategy. She explained:

I feel like I wouldn't want people to know that [I use drugs]. Like there was a point when I was in class like I wasn't getting clean works because I was so embarrassed to go in [to an SSP] and my counselor was like "That's stupid. That's part of harm reduction!”, but like I just felt so embarrassed like going in there.

Billy, who was not engaged in SSP services, explained how the shame he felt about his drug use prevented him from reaching out to people he trusted.

Q: So, when you [use] by yourself, did you ever have a check-in plan? What we mean by that is you call a friend and say “Hey, I’m about to use. Can you give me a call in another 10 minutes?”

Billy: No, no, never. Never. That’s not a bad idea, but, no, never.

Q: And why do you think you haven’t?

Billy: Because I’m trying to keep it a secret, so why am I going to promote it?

Q: But you do know people who use, but you still don’t feel like you could call them for stuff like that?

Billy: Yeah, but those people aren’t going to come rushing to my safety. That’s what I’m saying. I realize that those people were just in it for either financial benefit or a free ride or just-- we’re not really friends. We just had a mutual habit. The friends that’ll come rushing to my safety are the friends that I’m trying to keep it from.

## Discussion

Access to risk-reduction tools is imperative for people who use drugs. In this study, SSP participants had better access to naloxone and FTS and employed risk-reduction strategies such as check-in plans more frequently than non-SSP participants. This could be reflective of the success that SSPs have had in providing a non-stigmatizing space where PWUD are supported in adopting strategies to reduce risks related to their use [8]. These findings could also be indicative of the effectiveness of health education and promotion which, as previously noted, was the most common service provided by NYC SSPs in 2018 [8]. This positive influence of SSPs is particularly important during a time when opioid-involved overdoses are at historically high rates due to the increase in fentanyl in the drug supply.

Our findings suggest that unique and innovative approaches may be needed to increase the adoption of risk-reduction practices among those who are not engaged in SSPs. An example of such an approach is Never Use Alone [24], a toll-free number that connects PWUD to a person who will remain on the phone while they use and instigate a response should they experience an overdose. This service is particularly vital to those who are unable or unwilling to create a check-in plan with someone known to them, including those who may be less likely to be connected to a network of peers. Never Use Alone could potentially also mitigate feelings of shame and stigma by providing a non-judgmental service where people can maintain their anonymity.

Findings from this research also point to the need to expand access to and awareness of FTS among those not engaged in SSPs. For example, in an effort to reach a broader range of PWUD, NYC DOHMH provides free FTS to a range of community organizations (e.g., bookstores, nightlife venues), as well as services that intersect with people who use drugs. Additionally, supporting community-based organizations with an existing outreach infrastructure to raise awareness of fentanyl and promote FTS and overdose prevention education in the communities they serve may help expand their use.

Similarly, enabling easy access to naloxone for those not engaged in SSPs and promoting its benefits continues to be an important strategy. Findings from this study indicated that SSP participants witnessed more overdoses than those not engaged in SSP services, and previous research has demonstrated a positive association between witnessing an overdose and the adoption of risk-reduction practices [25]. Public health messaging, such as the “I Saved a Life” campaign which urges all NYC residents to carry naloxone, is essential to normalize this life-saving medication [26] and reduce the stigma that some associate with carrying naloxone.

Our findings, which suggest that those not engaged in SSP services were more resistant to carrying or using naloxone and, in some instances, held negative beliefs about naloxone, also highlight the importance of overdose prevention centers (OPCs), such as OnPoint NYC which opened in November 2021 [27]. Many OPCs, including OnPoint, use oxygen as a first-line response to potential overdoses and early intervention can prevent the need for naloxone administration. That OPC staff are appropriately trained to respond to symptoms of overdose might encourage people who have not yet engaged in OPC services to use the sites, especially if they know that naloxone will not be administered unnecessarily.

Of concern is the perception among some participants that intranasal use mitigates the risk of overdose. Recent data from the Relay initiative in NYC, a non-fatal overdose response system that offers peer support to individuals transported to the emergency department following an overdose event, found that only 23 percent of their participants reported injecting the drugs that resulted in their overdose [28]. This suggests that intranasal use still poses a significant risk of overdose and further highlights the need to reach those not engaged in SSPs who, in this study, were more likely to be using drugs intranasally. Further research into differences in the perceived risk of overdose between those who inject and those who use intranasally is warranted.

## Limitations

While this study had several strengths, such as the inclusion of those who do not access SSPs and a mixed-methods approach, there were notable limitations. First, the sample size was relatively small to assess for quantitative differences between those who accessed SSP services and those who did not. Second, there was some variability in the use of SSP services among those included in the SSP participant group. For example, some frequented static brick and mortar SSP sites on a regular basis, while others may have just obtained supplies (e.g., FTS, naloxone, or syringes) from a mobile site. This may have resulted in variability in access to tools to prevent fatal overdoses, support from other people who use drugs, and acquisition of information related to risk reduction from SSP sites. In addition, given that many of the participants not engaged with SSPs learned about FTS from participation in this study, social desirability may have played a role in the positive responses related to the potential utilization of FTS among this subgroup. However, members of this group also openly discussed their resistance to adopt other risk-reduction measures (e.g., carrying naloxone), suggesting that they were comfortable giving their opinions.

Lastly, the research team encountered challenges recruiting non-SSP participants as these individuals were less likely to congregate in settings where they could be identified as people who use drugs. While snowball sampling facilitated recruitment among this group, those who were not engaged in SSPs referred other participants less frequently compared to SSP participants.

## Conclusion

Our findings indicate that PWUD who are not engaged with SSPs have less access to and are less inclined to use some of the tools that could help reduce their risk of fatal overdose (e.g., FTS, naloxone, and check-in plans). Public health professionals, social workers, and others who engage with this population should encourage the adoption of these risk-reduction strategies as a means to reduce opioid-involved overdoses among this group. In addition, advocacy efforts could focus on exploring the potential of a safe drug supply through prescribing opioids (e.g., injectable diacetylmorphine or hydromorphone) to reduce the use of unregulated opioids, criminal activity, and the risk of fentanyl-related overdoses and deaths. Lastly, this study demonstrates that SSPs are providing life-saving tools to their participants and should be preserved and protected as a vital component to reduce overdose deaths.

## Data Availability

The datasets used and/or analyzed during the current study are available from the corresponding author upon reasonable request.
